# Special Issue “New Insights into Contraception”

**DOI:** 10.3390/jcm11226651

**Published:** 2022-11-09

**Authors:** Giuseppe Benagiano, Francesco M. Primiero

**Affiliations:** 1 Faculty of Medicine and Dentistry, Sapienza University of Rome, Policlinico Umberto I, Viale del Policlinico 115, 00161 Rome, Italy; 2 Faculty of Medicine and Psychology, Sapienza University of Rome, Ospedale Sant’Andrea, Via di Grottarossa 1035/1039, 00189 Rome, Italy

Today, a diverse range of contraceptive techniques is available to women; this, coupled with continued positive trends in female children and adults’ educational attainment, will hasten declines in fertility and continue to slow population growth. With the advent of a sustained fertility rate lower than the replacement level in many countries, including China and India, we are already witnessing major economic, social, environmental, and geopolitical consequences [1].

Hormonal contraception, which was initially available as a daily pill, can be administered through seven different routes today: intramuscularly, intranasally, intra-uterus, intravaginally, orally, subcutaneously, and transdermally. New advancements have also been made in intrauterine contraception with the development of “frameless” devices. Last, but not least, research is being undertaken to provide effective emergency contraception after unprotected intercourse. The use of selective progesterone receptor modulators (antiprogestins) is very promising in this area [2].

The expansion of contraceptive use worldwide was a main priority during the second half of the twentieth century. As countries in the developed world (but also in a number of developing countries) reached and even went below the replacement level of fertility, issues regarding contraception seem to have been placed on the back burner.

Yet, since the milestone final declaration of the Cairo Conference on Population and Development in 1994 [3], contraception has been considered the main tool used for effective family planning, while at the same time, too much emphasis has been placed on ‘technology’ as the solution. Unfortunately, each advancement in technology has shown that every new or improved method carries side effects which are disturbing to some users. Clearly, there is no perfect method. In addition, at the field level, national and regional family-planning programs are far from being ideally run. This is why the utilization of new technology will depend on the way it is applied in the field. Focusing on the needs of women (and men) will remedy the errors of the past, when contraceptive development was exclusively driven by scientists, without the consideration of users’ perspectives.

Because of this reality, the universal acceptance of methods of family planning, the best measure to prevent voluntary interruption of pregnancy (VIP), necessitates an integrated approach. Contraception must therefore be included in the broader concept of Sexual and Reproductive Health. This means that family planning and contraception must be placed within the wider paradigm of health, the health of the mother, and the health of her child. This in turn must be accepted as a task for individual couples, being a health and development issue, not a demographic one.

Promoting the use of contraception within the framework of improving health, not demography, means placing the emphasis on individuals, not methods alone, and on quality of services, not simply on availability through better distribution. Indeed, there is increasing awareness that contraception can help women to reduce health risks associated with reproductive events and specifically with unwanted pregnancies. In conclusion, no matter how ideal a method may be considered from a purely scientific viewpoint, it will be challenged by the constraints of the system in which it will be applied.

To contribute to the new *user-centered* perspective, the present issue of the *Journal of Clinical Medicine* presents a series of articles in which technology is considered within the broader concept of women’s health.

To begin with, five articles deal with issues related to users’ and providers’ perspectives. The first reported on a National Contraception Survey conducted to explore the sexual and contraceptive habits of 1801 Spanish women of reproductive age [4]. Among those enrolled in the study, 78.7% used some kind of contraceptive method; the most frequently utilized were condoms (31.3%) and combined oral contraceptives (COCs) (18.5%). Interestingly, one fourth of the participants used both condoms and a COC; the majority of these participants were younger women and those with no steady partners. The investigation found that health professionals have an important role in the choice of a method, although less so with teenagers. Finally, the survey found that emergency contraception (EC) is increasingly being used, especially among those not using contraception on a regular basis.

With regard to EC, a second investigation [5] tried to gather information regarding the profiles of users in Catalonia, Spain, finding that 44.2% of respondents were ‘repeat’ users, and among them, 56.7% used a barrier method; more than 40% of couples who had unprotected intercourse stated that they utilized a natural family-planning (NFP) method. The study concluded that there was still a high proportion of repeat users with risky sexual behaviors, highlighting the need to provide better information, education, and preventive strategies.

A third survey also from Spain [6], analyzed knowledge, attitude, and awareness towards EC among 478 nursing students at the University of Seville and found that all of them knew of the existence of EC and had a positive attitude towards this method. One fourth of responders had used the method, mainly because of condom failure, or because they did not use any form of contraception. The survey found improper knowledge of the mechanism of action, efficacy and repeated use, and the type of pills available. The authors concluded that further education initiatives should focus on the mode of use, efficacy, and mechanism of action.

Access to reproductive health and family-planning services during lockdowns in the COVID-19 pandemic was the subject of the fourth descriptive, cross-sectional study conducted in the Canary Islands (Spain) on a random sample stratified by age on a total of 1800 women (response rate: 98.72%) [7]. Given the difficulty of providing counseling in person, a telematic approach was also offered. This method did create access problems in one third of the cases, but it was considered an overall success, as it resulted in a considerable decrease in the recurrent use of both EC and services for the VIP. The survey evidenced the existence of cases of gender-based violence among women cohabitating with their aggressors during lockdowns.

The fifth report deals with an important question from users’ perspective of utilizing a contraceptive, namely the possibility that a given method (be it hormonal, barrier, or intrauterine) may modify a woman’s sexuality [8]. To explore this issue, the study provided an overview of the effects of different methods of contraception on female sexuality. It also summarized recent investigations in which a variety of positive and negative consequences of contraceptive use were described regarding several aspects of sexual functions (desire, arousal, orgasm, pain, and enjoyment), stressing that sexual satisfaction depends on factors that extend beyond sexual functioning alone.

Three of the published articles dealt with a modality that is gaining increasing amounts of attention: the so-called Long-Acting Reversible Contraception (LARC).

The first article presented the results of a retrospective electronic chart review of 311 insertions of either the levonorgestrel-releasing intrauterine device (LNG-IUS), or the subcutaneous etonogestrel implant (SEI) in a center in the USA [9]. Delays in insertion were found in 38% of the subjects, mostly because of the absence of a qualified provider or device availability. Teenage users favored the SEI, whereas older women preferred the LNG-IUS. Retention time varied by device type, and most subjects eventually switched to other contraceptives. No patients experienced expulsion of the system.

Some LARC methods, specifically the LNG-IUS, have been used for a variety of non-contraceptive indications; this was the subject of a review of the utility of the system in women with heavy menstrual bleeding and/or dysmenorrhea [10]. Data from the literature show accumulating evidence that the insertion of such a device represents a useful option for long-term treatment. As a consequence of bearing the system, there is an improvement in the quality of life, a reduction in menstrual blood loss superior to that obtainable with other medical therapies, and a decrease in the extent of dysmenorrhea and pelvic pain. The likely mechanism of action of the system seems to be its ability to induce amenorrhea, which effectively eliminates both symptoms.

An uncommon but important limitation in the use of the SEI is represented by social and psychiatric disorders, in which the ready accessibility of the device by the subject becomes a negative feature. To obviate this situation, a device was successfully inserted into the scapular region in a young woman with a chronic psychiatric disorder [11].

The last three contributions to this Special Issue dealt with new advancements in oral hormonal contraception (OHC).

An important unwanted effect in this field is bleeding irregularities, which is cited as one of the major reasons for the discontinuation of these methods, and therefore, bleeding irregularities have been the focus of numerous investigations. The first of the three articles regarding OHC in this issue provided an overview of bleeding data related to the recently marketed cyclic COC and one progestin-only pill (POP) [12]. In this study, data from recent trials carried out to gain regulatory approval was evaluated, and it was concluded that each type of OHC has its own specific bleeding pattern; unfortunately, methodological differences hampered a comparison between products. The first consideration is that the balance between the effects of progestin and estrogen on the endometrium, the key factor in producing a regular bleeding pattern, seems lost when using a too-low dose of ethinylestradiol (EE) in order to reduce the risk of venous thromboembolism. The recent attempt to replace EE by 17β-estradiol (E2) or E2 valerate may lead to a suboptimal bleeding profile due to endometrial destabilization. In an attempt to, if not eliminate, then at least decrease the occurrence of bleeding problems, it has been recently proposed to utilize a derivative of estriol, namely estetrol (15α-hydroxyestriol) (E4), a natural estrogen exclusively produced in the fetal liver, in combination with drospirenone (DRSP). This combination yields a predictable, regular bleeding profile. It is worth noting that when DRSP is employed alone, the resulting bleeding pattern becomes somewhat unpredictable, stressing the usefulness of adding an estrogen.

The issue of irregular bleeding patterns in POPs was discussed in the second article on OHC [13]. Starting from the fact that no effective solution to this problem has been found, the authors examined therapeutic options being offered by health providers through a prospective questionnaire comparing the effectiveness of various treatments. Different regimens were tested: (1) POPs with norethisterone; (2) double-dose POPs; (3) single-dose POPs; (4) different POP formulae. They found that women for whom 5 mg of norethisterone acetate was added reported a significant decrease in bleeding frequency compared to the other groups. This was associated with an overall decrease in bleeding quantity and frequency.

The last article in the series summarized the present state of a COC in which DRSP is combined with E4 [14]. Clinical studies show efficacy, bleeding patterns and tolerability comparable to those of preparations with EE, thus determining high acceptability and user satisfaction. In addition, minimal effects have been found on lipids, liver parameters, Sex Hormone Binding Globulin (SHBG), and carbohydrate metabolism. A possible lower risk of venous thromboembolism and a better cardiovascular safety profile need to be investigated in further, large-scale studies.

In conclusion, the field of contraception continues to expand in search for more acceptable and safer modalities and routes of administration. At the same time, today, increased attention is paid to users’ perspective and needs. This new focus means the available methods must be made suitable for different categories of subjects, ranging from teenagers to pre-menopausal women.

Today, contraception is much more than a technology; it has been the major instrument of a true social revolution that led to a—now often unwelcome—continued low fertility rate, while at the same time enhancing female reproductive health. Finding a correct balance between these aspects will be crucial in the years to come.
